# Developing an EEG-based on-line closed-loop lapse detection and mitigation system

**DOI:** 10.3389/fnins.2014.00321

**Published:** 2014-10-13

**Authors:** Yu-Te Wang, Kuan-Chih Huang, Chun-Shu Wei, Teng-Yi Huang, Li-Wei Ko, Chin-Teng Lin, Chung-Kuan Cheng, Tzyy-Ping Jung

**Affiliations:** ^1^Department of Computer Science and Engineering, Jacobs School of Engineering, University of California San DiegoLa Jolla, CA, USA; ^2^Swartz Center for Computational Neuroscience, Institute for Neural Computation, University of California San DiegoLa Jolla, CA, USA; ^3^Department of Electrical Engineering, National Chiao-Tung UniversityHsinchu, Taiwan; ^4^Department of Bioengineering, Jacobs School of Engineering, University of California San DiegoLa Jolla, CA, USA; ^5^Department of Biological Science and Technology, National Chiao-Tung UniversityHsinchu, Taiwan; ^6^Center for Advanced Neurological Engineering, Institute of Engineering in Medicine, University of California San DiegoLa Jolla, CA, USA

**Keywords:** electroencephalogram (EEG), drowsiness, fatigue, driving, smartphone, cell-phone, brain computer interface (BCI)

## Abstract

In America, 60% of adults reported that they have driven a motor vehicle while feeling drowsy, and at least 15–20% of fatal car accidents are fatigue-related. This study translates previous laboratory-oriented neurophysiological research to design, develop, and test an On-line Closed-loop Lapse Detection and Mitigation (OCLDM) System featuring a mobile wireless dry-sensor EEG headgear and a cell-phone based real-time EEG processing platform. Eleven subjects participated in an event-related lane-keeping task, in which they were instructed to manipulate a randomly deviated, fixed-speed cruising car on a 4-lane highway. This was simulated in a 1st person view with an 8-screen and 8-projector immersive virtual-reality environment. When the subjects experienced lapses or failed to respond to events during the experiment, auditory warning was delivered to rectify the performance decrements. However, the arousing auditory signals were not always effective. The EEG spectra exhibited statistically significant differences between effective and ineffective arousing signals, suggesting that EEG spectra could be used as a countermeasure of the efficacy of arousing signals. In this on-line pilot study, the proposed OCLDM System was able to continuously detect EEG signatures of fatigue, deliver arousing warning to subjects suffering momentary cognitive lapses, and assess the efficacy of the warning in near real-time to rectify cognitive lapses. The on-line testing results of the OCLDM System validated the efficacy of the arousing signals in improving subjects' response times to the subsequent lane-departure events. This study may lead to a practical on-line lapse detection and mitigation system in real-world environments.

## Introduction

Fatigue-related performance decrements such as lapses in attention and slowed reaction time could lead to catastrophic incidents in occupations ranging from ship navigators to airplane pilots, railroad engineers, truck and auto drivers, and nuclear plant monitors. Fatigue (or drowsiness) “concerns the inability or disinclination to continue an activity, generally because the activity has been going on for too long,” defined by European Transport Safety Council (Croo et al., 2001). Sixty percent of American adults reported that they have been driving a motor vehicle when feeling drowsy (National Sleep Foundation, 2005). Furthermore, studies have concluded that at least 15–20% of fatal car accidents are fatigue-related (The Royal Society for the Prevention of Accidents, 2001; Connor et al., 2002; National Highway Traffic Safety Administration, 2003). Therefore, an earlier detection of driving fatigue is a crucial issue for preventing catastrophic incidents.

In order to detect the driving fatigue, several approaches have been proposed in scientific literature. (1) Computer vision-based systems (Bergasa et al., 2006; D'Orazio et al., 2007; Golz et al., 2010). Bergasa et al. (2006) used a real-time image-acquisition system to monitor drivers' visual behaviors that revealed a drivers' alertness level. Six parameters: percentage of eye closure, eye closure duration, blink frequency, nodding frequency, face position, and fixed gaze were included in a fuzzy classifier for identifying a driver's vigilance level. D'Orazio et al. (2007) proposed a neural classifier to recognize the eye activities from images without being constrained to head rotation or partially occluded eyes. (2) Driving behavior counter-measurements (Lin et al., 2009, 2010a, 2013). Lin et al. (2009) performed an event-related, lane-keeping driving task in an immersive virtual-reality environment. Subjects were asked to steer the stimulated car back to the middle of the cruising lane once they perceived the randomized lane-departure events. The results showed that the reaction time (RT), defined as the time interval between the onset of the simulated car deviation and the user response, could be improved by providing arousing auditory warning to the subjects combating with fatigue.

A Brain Computer Interface (BCI) translates neural activities into control signals to provide a direct communication pathway between the human brain and an external device (Wolpaw et al., 2002). Broadly speaking, BCIs can be grouped into three categories: active, passive and reactive BCIs (Zander and Kothe, 2011). Electroencephalogram (EEG)-based passive BCIs measure brain electrical activities from the scalp and enrich a human–machine interaction with implicit information on the actual user state without conscious effort from the user (Lehne et al., 2009; Zander et al., 2009, 2010; Zander and Jatzev, 2012). Given appropriate signal-processing algorithms in the passive BCIs, meaningful information can be directly extracted from the EEGs. For instance, time-domain analysis such as averaging across different channels, moving average with a specific window length, standard deviation, linear correlation and so on are useful approaches to extract information from EEGs (Dong et al., 2011). In a frequency-domain analysis, the short-time Fourier transform (STFT) is often applied to the EEG data to estimate the power spectral density in distinct frequency bands, including delta (1–3 Hz), theta (4–7 Hz), alpha (8–13 Hz), beta (14–30 Hz), and gamma (31–50 Hz). Many studies have shown that the brain dynamics linked to fatigue and behavioral lapses can be assessed by EEG power spectra (Kecklund and Akerstedt, 1993; Makeig and Inlow, 1993; Jung and Makeig, 1995; Makeig and Jung, 1996; Jung et al., 1997; Lal and Craig, 2002; Campagne et al., 2004; Horne and Baulk, 2004; Debener et al., 2005; Peiris et al., 2006; Davidson et al., 2007; Eichele et al., 2010, 2008; Golz et al., 2010; Lin et al., 2010a), combinations of EEG band power (Jap et al., 2011), alpha spindle parameters (Simon et al., 2011) and autoregressive features (Rosipal et al., 2007). These studies provided solid evidence for the neurophysiological correlates of fatigue and behavioral lapses. In short, while the physical- and behavioral symptom-based methods indirectly measure drivers' cognitive states, the neurophysiology-based methods offer a more direct path to assess the brain dynamic linked to fatigue and behavioral lapses with a high temporal resolution.

Efforts have also been made to assist individuals in combating fatigue and/or preventing lapses in concentration. For instance, Dingus et al. (1997) and Spence and Driver (1998) proposed using warning signals to maintain drivers' attention. The types of warning signals could be auditory (Spence and Driver, 1998; Lin et al., 2009), visual (Liu, 2001), tactile (Ho et al., 2005) or mixed (Liu, 2001). Empirical results showed that auditory warning could reduce the number of lapses in sustained-attention tasks (Spence and Driver, 1998), and could help subjects to maintain driving performance (Lin et al., 2009). More recent studies demonstrated that arousing auditory signals presented to individuals experiencing momentarily behavioral lapses could not only agitate their behavioral responses but also change their EEG theta and alpha power in a sustained-attention driving task (Jung et al., 2010; Lin et al., 2010a, 2013). However, the studies also showed that sometimes subjects did not respond to the arousing signals, and more importantly the EEG activity of these non-responsive episodes showed little or no changes following the ineffective warning (Jung et al., 2010; Lin et al., 2010a, 2013). Lin et al. (2013) later demonstrated the feasibility of using the post-warning EEG power spectra to predict the (in)efficacy of the arousing warning. A caveat of their studies was that the arousing warning was delivered to subjects after they behaviorally failed to respond to lane-departure events. In reality, the delivery of arousing warning could have been too late because the behavioral lapse might have led to catastrophic consequences. A truly EEG-based lapse monitoring system needs to continuously and non-invasively observe EEG dynamics to predict fatigue-related lapses, deliver arousing signal to arouse the user, and assess the efficacy of the arousing signal to trigger a repeated or secondary warning signal if necessary. Furthermore, all of the aforementioned studies were conducted with traditional bulky and tethered EEG systems and were performed in well-controlled laboratories. However, it is argued that there might be fundamentally dynamic differences between laboratory-based and naturalistic human behavior in the brain (McDowell et al., 2013). It thus remains unclear how well the current laboratory-oriented knowledge of EEG correlates of cognitive-state changes can be translated into the highly dynamic real world.

This study aims to extend previous studies to design, develop and test a truly On-line Closed-loop Lapse Detection and Mitigation (OCLDM) System that can continuously monitor EEG dynamics, predict fatigue-related lapses based on EEG signals, arouse the fatigued users by delivering arousing signals, and assess the efficacy of the arousing signal based on EEG spectra. This study hypothesized that (1) EEG spectral values would differ under different arousal states; (2) it is feasible to predict lapses based on the spectral changes in the spontaneous EEG; (3) arousing warning delivered to cognitively challenged subjects would mitigate cognitive lapse, and (4) the rectified performance would be accompanied by the changes in EEG power spectra. This study conducted an off-line experiment to explore the neurophysiological correlates of lapses, which tested the abovementioned hypotheses and guided the development of a truly OCLDM system. The system was then validated by an on-line driving experiment. Furthermore, to be practical for routine use in a car or workplace by freely moving individuals, the EEG-based lapse monitoring system must be non-invasive, non-intrusive, lightweight, battery-powered, and easily to put on and take off (Lin et al., 2008a,b). This study thus also investigates the feasibility of using a practical, low-density, lightweight dry EEG headgear and a smartphone-based EEG-processing platform (Wang et al., 2012) to build a truly mobile and wireless OCLDM System for real-life applications.

## Materials and methods

### Subjects

Eleven healthy and naive subjects (10 males and one female) with normal hearing and aged 20–28 years old participated in this study. All of them were free of neurological and psychological disorders. They were introduced how to manipulate the car and practiced ~10 min to get acquainted before the experiment started. None of them worked night shifts or traveled across multiple time zones in the previous 2 months. All participants were asked to read and sign the informed consent form before participating in the studies. After the experiments, subjects were asked to complete the questionnaire for assessing their cognitive states during the experiments.

### Experimental equipment

Experiments of this study were conducted in an 8-screen and 8-projector immersive virtual-reality (VR) environment that simulates the 1st person view scene of highway driving. This study adapted an event-related lane-departure driving paradigm originally proposed by Huang et al. (2006, 2007a,b, 2009) that allowed objective and quantitative measures of momentary event-related brain dynamics following lane-departure events and driving-performance fluctuations over longer periods. The VR scenes simulated driving at a constant speed (at ~100 km/h) on a highway with the simulated car randomly drifting away from the center of the cruising lane to simulate driving on non-ideal road surfaces or with poor alignment (Huang et al., 2007a,b, 2009; Lin et al., 2008b, 2009). The scene was updated according to the land-departure events and the subject's manipulation. The vehicle trajectory, user's input, and lane-departure events could be accurately logged and time-synchronized to the EEG recordings (Huang et al., 2007a,b, 2009; Lin et al., 2008b). There were no traffic or distractive objects other than 4-lane roads and dark sky appeared in the VR while the simulated car was cruising on the highway.

Thirty-two channel EEG data were collected from participants by the NuAmp system (32-channels Quick-Cap, Compumedics Ltd., VIC, Australia). The electrodes were placed according to a modified international 10–20 system with a unipolar reference at the right earlobe. The EEG activities were recorded with 500 Hz sampling rate and 16-bit quantization level.

### Experimental paradigm

**Figure 1:**
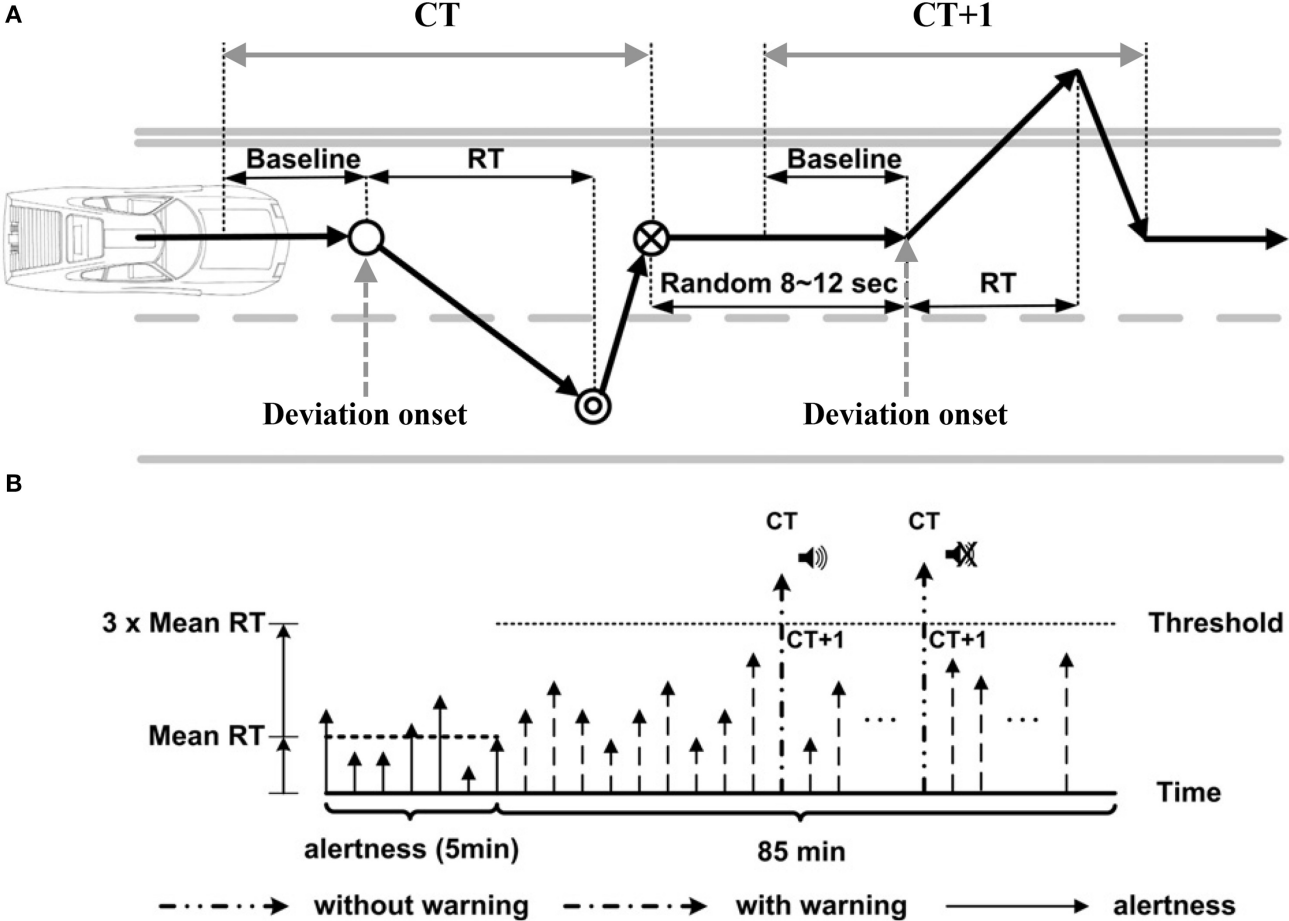
**Experiment paradigm. (A)** Event-related lane-departure driving tasks. The solid arrows represent the driving trajectory. The empty circle represents the deviation onset. The double circle represents the response onset. The circle with the cross represents the response offset. The baseline is defined as the 3 s period prior to deviation onset. The response time (RT) of a driver is the interval from the deviation (empty circle) to the response onset (the double circle). A trial starts at deviation onset and ends at response offset (circle with a cross). The next deviation begins 8–12 s after response offset. **(B)** Criterion for delivering auditory warning during driving tasks. The height of an arrow represents the response time in a single trial. The warning was delivered to the subject when the RT in the trial exceeded three times the mean RT of trials in the first 5 min of the task, when the subject was presumably alert and fully attended to lane-departure events. In this figure is adapted with permission from Figure 1 of Lin et al. (2010a).

Figure 1A shows the experimental paradigm of this study. The simulated car starts cruising at a fixed speed (~100 km/h) on the 3rd lane and drifting to either right or left with equal probability within 8–10 s. Subjects were instructed to steer the simulated car back to the 3rd lane as soon as they noticed the lane drift. The simulated car keeps cruising on the right (or left) most lane if the subjects failed to respond to lane drift. The baseline period of each lane-departure epoch is defined as the 3 s before the onset of a lane-drifting event. The empty circle in Figure 1A represents the unexpected lane-departure events marked as the “deviation onset.” After the deviation onset, subjects were instructed to steer the simulated car back to the center of the cruising lane immediately (double circle), and the time when the subjects started steering was marked as the “response onset.” The moment that the simulated car reached the center of the cruising lane (circle with cross) was marked as the “response offset.” A subject's response time (RT) was defined as the time between the deviation onset and the response onset. At the first 5 min of the experiment, subjects were asked to be fully alert, verified by the vehicle trajectory and the video from a surveillance camera, to obtain an averaged alert RT (aRT) for each subject (1.51~2.54 s), which is a threshold for the entire experiment. The entire experiment consisted of 5 min training and 85 min driving periods.

Figure 1B shows the criterion of delivering auditory warnings in the experiment. When a subject failed to respond within three times the aRT, the system treated the trials as a behavioral lapse and triggered a 1750 Hz tone-burst to arouse the subject from fatigue-related lapse in half (50%) of these drowsy trials (marked as the “current trial (CT)” in Figure 1A). The very next trial is defined as CT + 1, and so on. The lapse trials that were randomly selected to receive arousing warning were referred to as CT with warning, whereas the remaining half of trials that did not receive auditory warning were referred to as CT without warning. Note that our previous studies showed that in some trials subjects remained non-responsive following the arousing warning, which was analogous to sleeping through an alarm clock (Jung et al., 2010; Lin et al., 2013). If the RT of the following trial (CT + 1) was shorter than the double of the averaged aRT, the warning signal delivered in the CT trial was defined as an “effective warning.” On the other hand, if the RT of the CT + 1 trial was longer than triple of the averaged aRT, the warning was defined as an “ineffective warning.” This study did not include the trials with RTs between 2 and 3 aRT to define the alert vs. fatigue spectral thresholds because the cognitive states of the subjects during those trials were unclear. Note that, subjects didn't know about the warning before the experiments.

### Data analysis

The 32-channel EEG data were first down-sampled to 250 Hz, and a low-pass filter of 50 Hz and a high-pass filter of 0.5 Hz were applied. Channels or trials with severe artifacts (such as body movements or muscle activities) were manually removed (less than three channels and 20% trials per subject in general). The remaining EEG data were segmented into several 115 s trials, each of them consisting of 15 s before and 100 s after the lane-deviation onsets. Independent Component Analysis (ICA, Bell and Sejnowski, 1995; Makeig et al., 1997) implemented in EEGLAB (Delorme and Makeig, 2004) was then applied to decompose the ~32-channel EEG into ~32 independent components (ICs), based on the assumption that the collected EEG data from the scalp were a weighted linear mixture of electrical potentials projected instantaneously from temporally ICs accounting for distinct brain sources. The comparable ICs across subjects were grouped into component clusters based on their scalp maps, equivalent dipole locations and baseline power spectra of component activations (Jung et al., 2001; Delorme and Makeig, 2004). Across 11 subjects, there were 155 trials with warning (30 trials were ineffective and 125 trials were effective) and 192 trials without warning.

Since the RT and EEG power were not normally distributed, non-parametric statistic tests were performed for the data analysis (Delorme and Makeig, 2004). The Wilcoxon rank-sum test (Matlab statistical toolbox, Mathworks) was used to assess the effects of warning on RTs. Bootstrapping (EEGLAB toolbox, University of California, San Diego) was used to test the statistical significance of EEG power changes at specific frequency bins from 2 to 30 Hz with a 0.25 Hz resolution. To test group statistics, the intrinsic inter-subject RT differences were reduced by dividing RTs by the mean RT. The EEG spectra were normalized by dividing the spectral power by the standard deviation of the spectral distribution.

## Results: neurophysiological correlates of behavioral lapses

### Efficacy of arousing auditory signals for rectifying lapses

**Figure 2:**
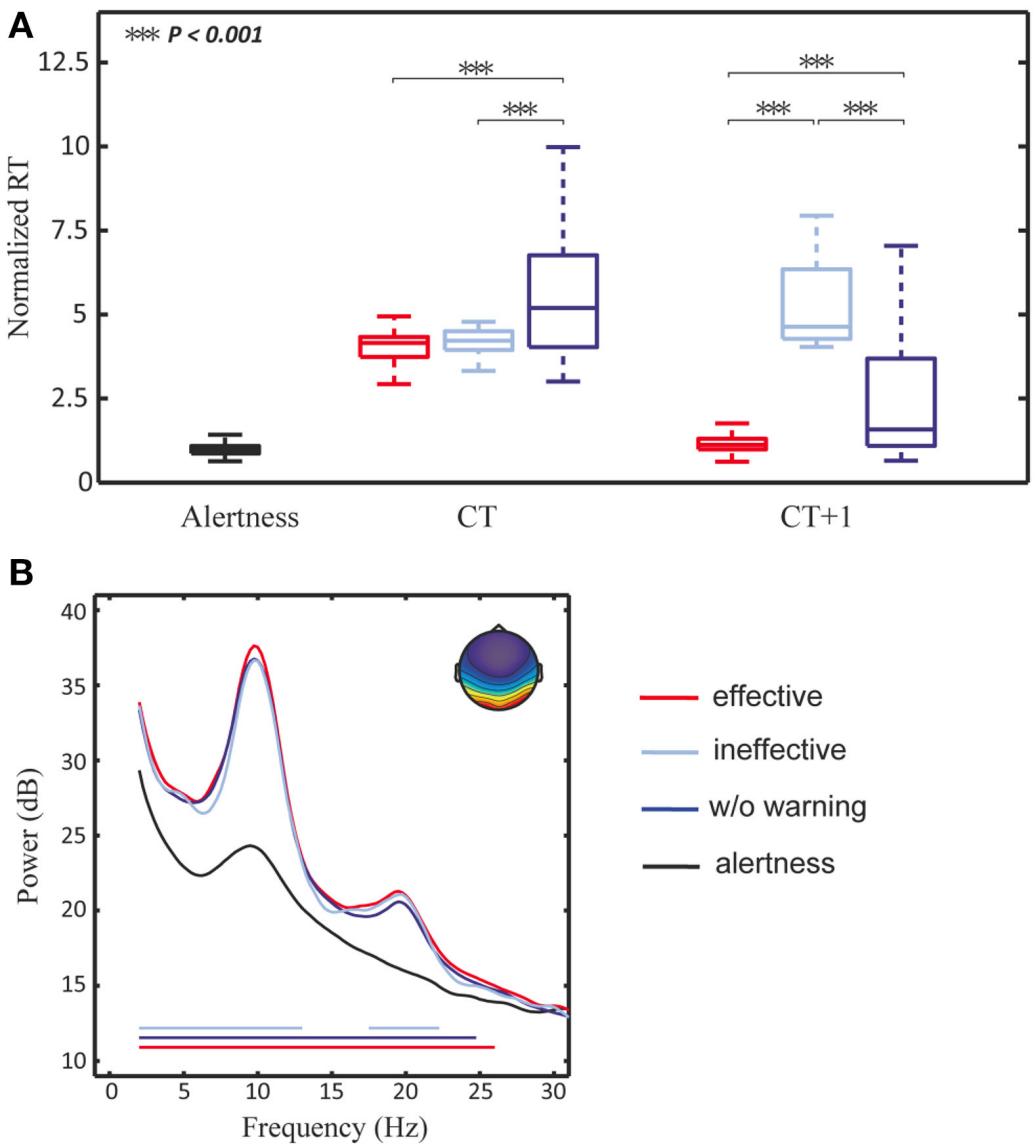
**Experiment results**. **(A)** The boxplot for the RT distribution of trials with effective warning, ineffective warning, and without warning among CTs and CTs + 1. Note that middle horizontal line is the median of the distribution, and the top and bottom of the rectangle are the third and first quartile, and the dash line ends are the maximum and minimum after outlier removal. **(B)** The component spectra of the alert CTs (black curve), with an effective warning (red curve), with an ineffective warning (light blue curve) and without warning (dark blue curve). The red, light blue and blue horizontal lines mark the spectral differences between the alert trials and trials with an effective warning, with an ineffective warning, and without warning, respectively. All the spectral plots were calculated from the activity of the bilateral occipital components separated by ICA.

This study first explored the efficacy of the delivery of arousing auditory signals by measuring the change in subjects' reaction time. Figure 2A shows the boxplots of RTs of three trial groups: Alertness, CT, and CT + 1 (left to right). The averaged aRT of trials within the Alertness group across 11 subjects was ~676 ms. The RTs of the CT group with arousing warning (red and light blue) were statistically significantly shorter than those of trials without receiving arousing warning (dark blue). The RTs of the CT + 1 group with effective vs. ineffective warning differed while the RTs of the preceding group (CT) were comparable. Even though the subjects responded to the arousing warning by immediately steering the simulated car back to the cruising position, they could well be totally non-responsive to the very next lane-departure event (~10 s later). In other words, the arousing signals reliably rectified human behavioral lapses, but did not guarantee that subjects were fully awake, alert, or attentive. This suggests an analogous regime of snooze after an alarm is turned off.

### EEG dynamics preceding behavioral lapses

Figure 2B shows the mean scalp map of the bilateral occipital cluster (upper-right corner) and its component baseline power of drowsy trials without auditory warning (dark blue), with either effective (red) or ineffective warning (light blue). First, among the resultant ICA clusters, bilateral occipital components exhibited statistically significant spectral differences between trials with and without auditory warning. Second, the component power spectra exhibited tonic increases in theta (4–7 Hz), alpha (8–12 Hz), and beta (13–30 Hz) bands in drowsy trials (red, dark blue, and light blue), compared to the alert trials (black). Horizontal lines mark the frequency bins under which the spectral differences between alert trials and drowsy trials with either (in)effective warning, or without warning were statistically significant (alpha = 0.05, Bonferroni adjusted *p*-value of 0.05/(112 frequency bins) = 0.0004 for multiple comparisons). Note that the spectra shown here were calculated from the component activities prior to the lane-deviation onset. The nearly identical pre-lapse spectra of these three groups of non-responsive trials demonstrate the robustness of the broadband spectral augmentation preceding the behavioral lapses, suggesting the feasibility of using theta and alpha power from the lateral occipital areas to predict behavioral lapses in this sustained-attention driving task.

### Effects of arousing auditory signals on the EEG

**Figure 3:**
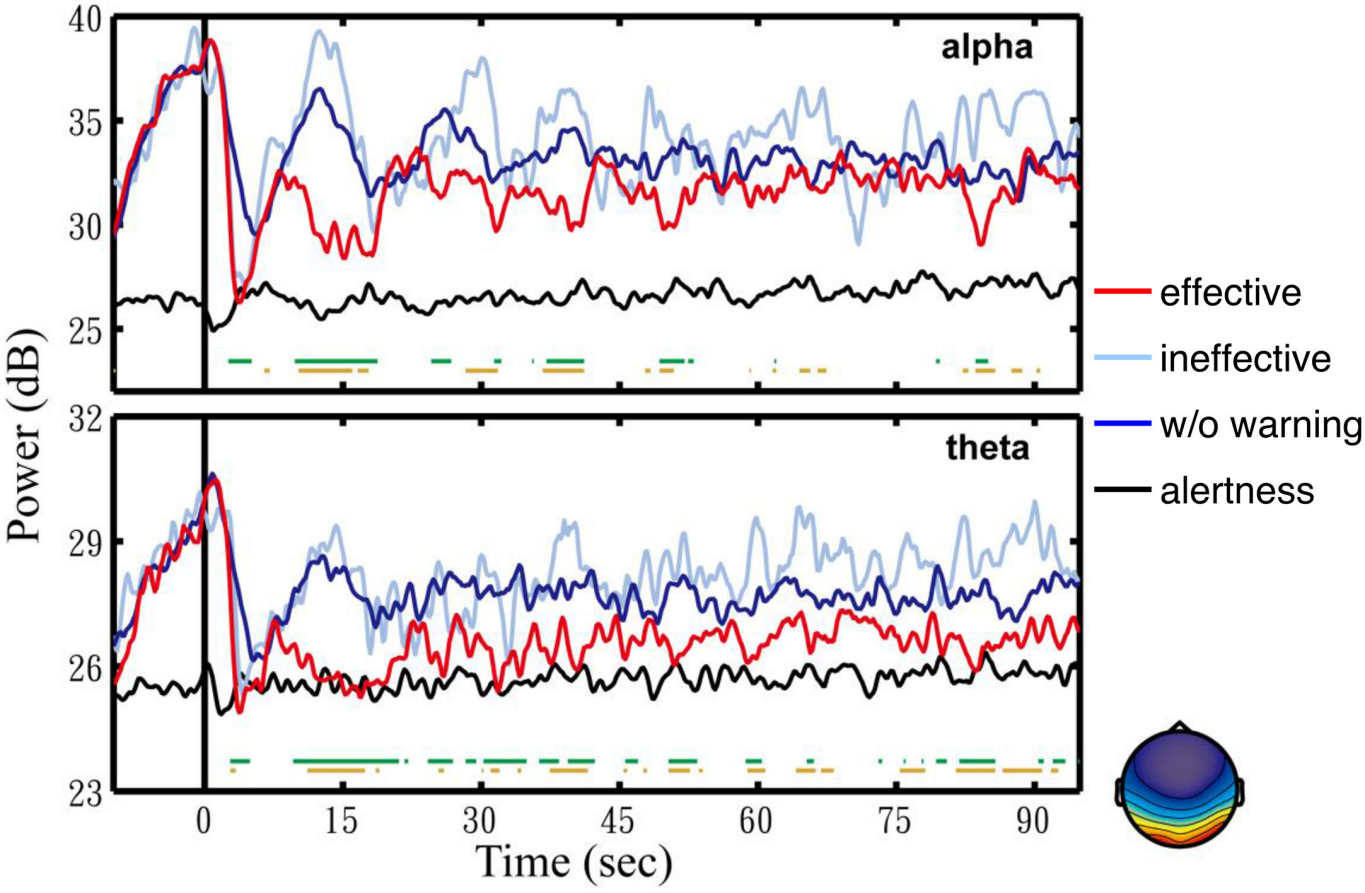
**Average component EEG power changes in alpha (top panel) and theta (bottom panel) bands from the bilateral occipital components (lower right corner)**. All the trials are aligned to the lane-deviation onsets at time 0 s (vertical solid black line). The red, light blue, dark blue, and black traces are the averaged spectra of trials with effective feedback, with ineffective feedback, without feedback, and in alertness, respectively. The green horizontal line indicates the statistically significant differences (*p* < 0.01) between trials with effective feedback and without feedback. The brown indicates the statistically significant differences (*p* < 0.01) between trials with effective feedback and ineffective feedback.

Next, this study explored temporal spectral dynamics preceding, during and following fatigue-related behavioral lapses and following arousing warning. Figure 3 shows time courses of spectral changes in the bilateral occipital area following ineffective warning (light blue trace), effective warning (red trace), and without warning (dark blue trace), compared to those of the alert trials (black trace). Figure 3 shows that both theta- an alpha-band power steadily increased prior to the lane-departure onset (at time 0 s). Again, the trends of steady increasing theta- and alpha-band power leading to behavioral lapses in the three groups of drowsy trials were nearly identical, indicating the robustness of the theta and alpha augmentation preceding the behavioral lapses.

Figure 3 also shows that after the lane-departure onset (at time 0 s), the alpha (top panel), and theta (bottom panel) power abruptly decreased by over 10 and 5 dB to nearly the alert (black trace) baseline, respectively. More importantly, following the subjects' responses, the spectra of trials with ineffective warning (light blue trace) and without warning (dark blue trace) rapidly rose from the alert baseline to the drowsy level in 5–15 s. The theta and alpha power of trials with effective warning, however, remained low for ~40 s. The green horizontal lines mark the time points when the difference between the spectra of trials with effective warning and without warning were statistically significant (*p* < 0.01). The spectral difference between the trials with effective warning and without warning was significant from 7 to 18 s in alpha band and from 7 to 21 s in the theta band (*p* < 0.01). Furthermore, the spectral difference between the trials with effective and ineffective warning was significant from 7 to 16 s in both alpha and theta bands (brown horizontal lines).

In sum, these results provided invaluable insights into the optimal electrode locations (lateral occipital region) and EEG features (theta- and alpha-band power) for a practical OCLDM system detailed below. The EEG and behavioral data collected from this experiment were used to assess the EEG correlates of fatigue-related lapses and build a lapse prediction model for the second experiment.

## Developing a OCLDM system

Our previous study (Wang et al., 2012) proposed a cell-phone based drowsiness monitoring and management system to continuously and wirelessly monitor brain dynamics using a lightweight, portable, and low-density EEG acquisition headgear. The system was designed to assess brain activities over the forehead, detect drowsiness, and deliver arousing warning to users experiencing momentary cognitive lapses, and assess the efficacy of the warning in near real-time. However, the system was not fully implemented nor experimentally validated in humans. Furthermore, according to the neurophysiological results in section Results: Neurophysiological Correlates of Behavioral Lapses, EEG signals collected over the lateral occipital regions were more informative for lapse detection. This study extends the previous work to design, develop, and test an OCLDM System.

### System architecture

**Figure 4:**
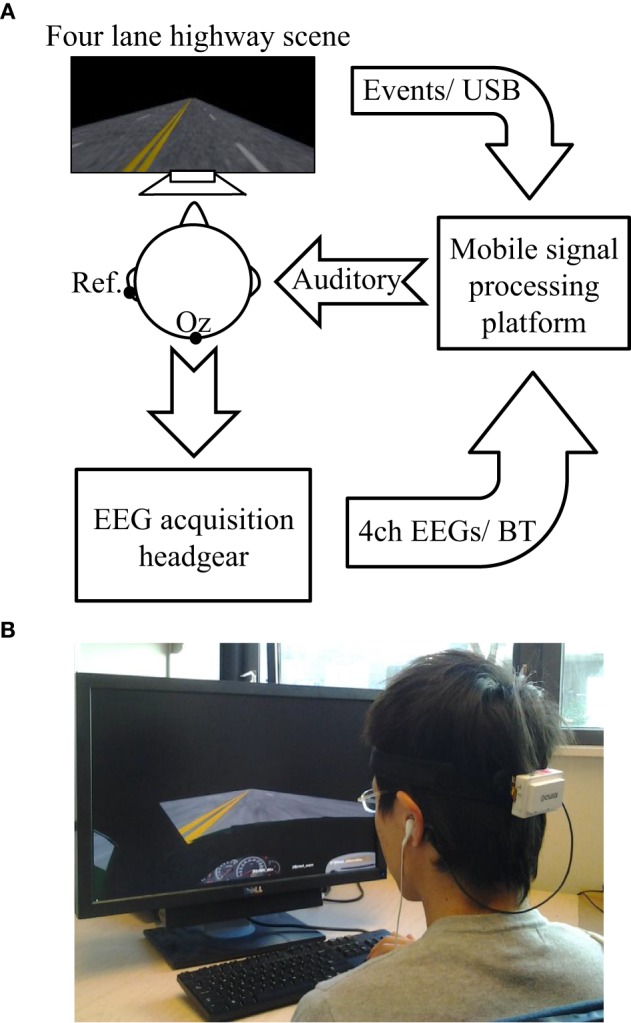
**The system diagram of the proposed OCLDM System**. **(A)** The EEG headgear collected 4-channel brain activities from the lateral occipital area while a subject was performing the lane-drifting experiment. The mobile signal-processing platform received the acquired EEG raw data through Bluetooth, and the event markers generated from the lane-departure scene through an USB interface. Finally, the auditory feedback was delivered to the subject when the averaged EEG power across four channels was 3 dB over the alert baseline. **(B)** A photo of a subject performing the on-line driving experiment while wearing a 4-channel EEG headgear (the white small box attached on a flexible band) over the lateral occipital area.

Figure 4A shows the system diagram of the proposed OCLDM System. The system consists of two major components: (1) a mobile platform featuring the OCLDM algorithm, and (2) a mobile and wireless 4-channel headgear measuring EEG signals over the hair-bearing occipital regions with dry EEG sensors (Liao et al., 2011). The OCLDM System was implemented as an App on an Android-based platform (e.g., Samsung Galaxy S3). The smartphone has a Bluetooth module, 16 GB RAM, an ARM Cortex-A9 processor, Android (Ice Cream Sandwich) OS, and other components. When the App is launched, it can automatically search and connect to a nearby EEG headgear to receive data from the EEG acquisition headgear. In the mean time, the App opened an USB port to receive the events from a four-lane highway scene to synchronize the EEG data and scene events. The build-in speaker (or plug-in a ear set) of the smartphone delivers auditory warning signal once the OCLDM System detects that the subject is experiencing a cognitive lapse. Both the EEG data and scene-generated events could be logged onto either smartphone's build-in memory or an external microSD card for further analysis.

The mobile and wireless EEG acquisition headgear features a 4-channel lightweight portable bio-signal acquisition device powered by a 3.7 v Li-ion battery (Lin et al., 2010b). It consists of a TI MSP430 microprocessor, a pre-amplifier, a battery-charging circuit, a 24 bit ADC, a Bluetooth module, and dry spring-loaded EEG sensors (Liao et al., 2011). The spring-loaded probes of the sensor can penetrate the hair to provide good electrical conductivity with the scalp. The microprocessor controls all the components including the amplifiers, digitizers, and transmits the digitalized EEG data to the Bluetooth module. The 4-channel EEG data are then transmitted to the authorized receiver of the OCLDM System. Depending on the applications, the system's sampling rate can be programmed at 128, 256, or 512 Hz. An experienced subject can easily put on this EEG acquisition device within 1–3 min without any help from a technician. Figure 4B shows a photo of a subject wearing a 4-channel EEG headgear and performing the simulated driving experiment.

### System software design

**Figure 5:**
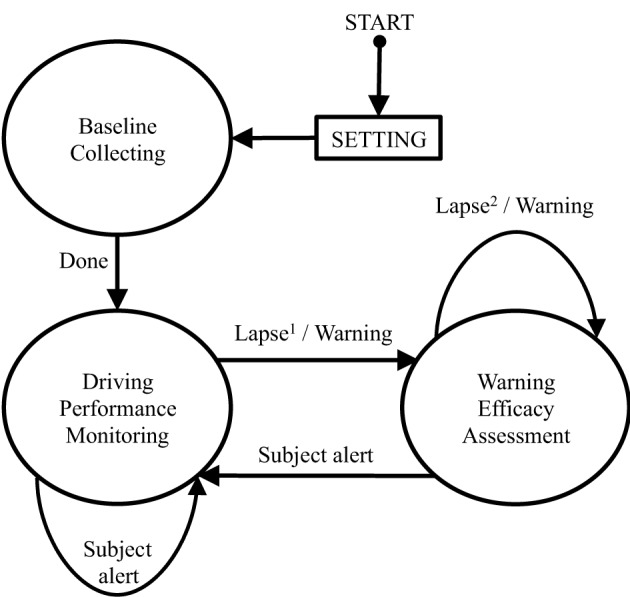
**The software state diagram of the OCLDM System**. The program first goes through SETTING and Baseline collecting state. Then, the system continuously detects and monitors subjects' driving performance until the EEG spectra indicate cognitive lapse. Note that, Lapse^1^ represents the averaged EEG power across four channels is 3 dB over the alert baseline. Lapse^2^ represents that the averaged EEG power across four channels has not yet dropped 3 dB from the lapse power.

Figure 5 shows the program's state diagram of the proposed OCLDM System. Three major states, including Baseline Collecting (BC), Driving Performance Monitoring (DPM), and Warning Efficacy Assessment (WEA), were implemented in the program. When the program is launched, one can modify the parameters in the SETTING page, shown as a square box in the figure. For instance, the parameters can be the duration of baseline data collection, or the threshold of auditory warning delivering for the other two states. Depending on the applications, the lapse threshold in the DPM state can be calculated accordingly. For example, one can use a combination of power of alpha, beta, theta, and delta bands to detect cognitive lapse. The program then enters the next (DPM) state after the Baseline (calibration) data collection has completed. The DPM module continuously monitors the driver's neurophysiological data. The program stays in the DPM state until the lapse threshold is met, which depends on the neurophysiological results as shown in section Results: Neurophysiological Correlates of Behavioral Lapses. For instance, when the subject's power spectrum in alpha band is 3 dB higher than the threshold (alert baseline collected in the BC state), the program delivers an auditory warning to arouse the subject and enters the FEA state. The current value is stored as the lapse reference in the FEA module. The system repeatedly delivers auditory warning until the EEG power decreases to another threshold.

### On-line experimental paradigm

Three new male subjects (who did not participated in the first experiment) with normal hearing and aged 25–30 years old participated in the on-line closed-loop lane-departure driving experiments to evaluate the OCLDM System in a more naturalistic setting (in a regular office without any electromagnetic shielding). All of them were asked to read and sign the informed consent form before participating in the studies.

The entire experiment consisted of a 1 min training and a ~60 min driving periods. During the training session, subjects were asked to stay fully alert. The averaged alpha power collected in the BC session was used as an alert baseline to determine whether a subject is experiencing cognitive lapses in the driving task. The subject performed the lane-departure driving experiments following the protocol below:

1. Subjects seated in an armchair and the driving scene was displayed on a 27″ monitor, placed at ~60 cm in front of the subject.
2. Subjects used a keyboard to control a vehicle cruising on a high way, i.e., a left key turns the simulated car to the left while a right key turns to the right.
3. Four electrodes were placed over the lateral occipital area to collect EEG data non-invasively. The data were transmitted to a smartphone for processing via Bluetooth.
4. When the averaged power spectra in alpha band met a certain criterion, arousing auditory warning (~65 dB 1750 Hz tone-burst) would be delivered in half of these lapse episodes through an ear set to the subjects. Note that, the subjects didn't know the warning before the experiments.
5. The arousing tone-burst would be continuously delivered to the subjects until the averaged power spectra in the alpha band has dropped 3 dB from the lapse power.

The cognitive lapses were detected when the subject's alpha-band power (Jung et al., 2010; Lin et al., 2010a, 2013), calculated by a moving-averaged STFT with a 256-point sliding window advanced at 1 s step running on the smartphone, was 3 dB over the alert baseline power (Lin et al., 2010a, 2013 and Results in section Results: Neurophysiological Correlates of Behavioral Lapses). This study used the alpha power fluctuations to monitor cognitive lapses because (1) a recent study showed that the alpha augmentation was sensitive to the transition from full alertness to mediate drowsiness, while the theta augmentation was more sensitive to the transition from mediate to deep drowsiness (Chuang et al., 2012); (2) the empirical results of this study showed that the augmentation of alpha-band power changes was greater than that of the theta-band power (Figure 2). The system would repeatedly deliver auditory warning until the alpha-band power amplitude has dropped to 3 dB below the power level when the cognitive lapse was identified.

### Results from the OCLDM system

The numbers of detected cognitive lapses varied across subjects. Table 1 lists the numbers of trials with effective, ineffective warning and without warning, respectively. Here, the way we defined the effective trials was based on the RT in response to the lane-departure event immediately following the arousing signal (CT + 1 whose RT was shorter than two times aRT); while the ineffective trials had RT longer than three times aRT.

**Table 1:** **Number of trials collected from the on-line experiment**.

	**With auditory warning**		**Without auditory warning**
	**Effective**	**Ineffective**	
Subject 1	20	2	21
Subject 2	17	1	24
Subject 3	23	0	27

**Figure 6:**
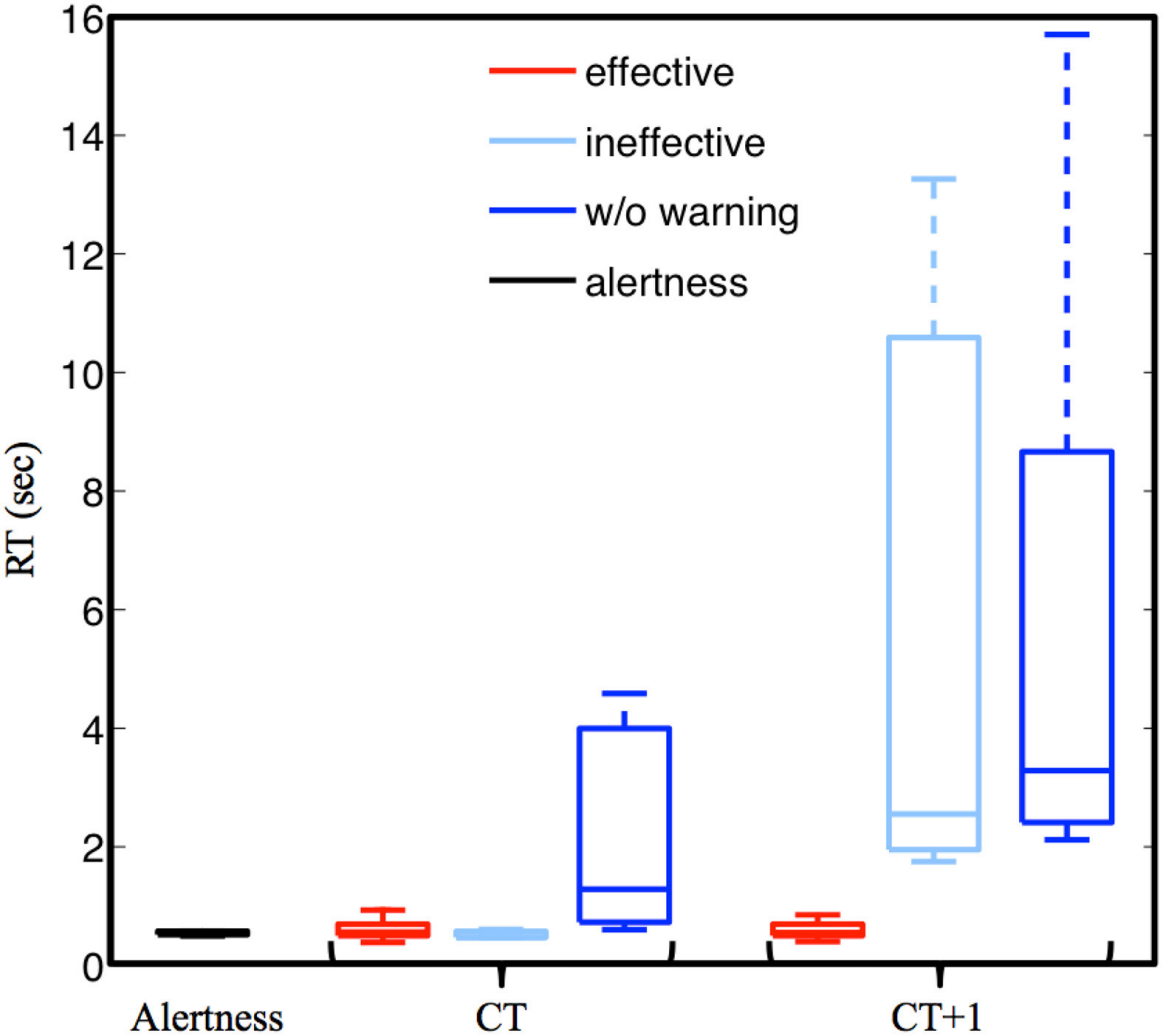
**The behavioral performance comparison for the trials of with effective feedback (red), with ineffective feedback (light blue), and without feedback (blue), compared to alert trials (black) after removing the outliers**.

Figure 6 shows the boxplot of behavioral performance (RTs) of trials with effective trials (red), ineffective trials (light blue), and without warning (dark blue), compared to the averaged aRT (black) during the on-line experiments. The effective trials had RTs comparable to the averaged aRT (less than 1 s) in both CT and CT + 1. Note that the RTs of CT + 1 with effective vs. ineffective warning differed largely because that was how the effective and ineffective trials were defined. However, the RTs of CT trials (red and light blue) of these two groups of trials were very comparable. That is, even though the subjects responded to the arousing warning by steering the simulated car immediately back to the cruising position, they could well be totally non-responsive to the very next lane-departure event. This finding is consistent to our off-line study reported in section Results: Neurophysiological Correlates of Behavioral Lapses in which the arousing warning was delivered to the subjects who just had a behavioral lapse.

**Figure 7:**
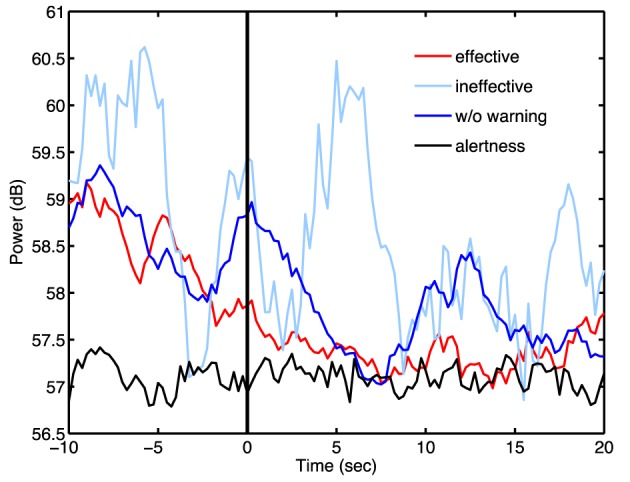
**The averaged alpha power time course plotting time-locked to subject response onset (vertical solid line at time 0 s)**. Averaged alpha power of trials with effective feedback (red trace), with ineffective feedback (light blue), and without feedback (blue trace), compared to trial with aRT (black). The time course of power was estimated by short time Fourier transform with 256 points of time window and 224 points overlapping.

Figure 7 showed the averaged alpha-band spectral time courses across subjects and trials with effective warning (red trace), with ineffective warning (light blue trace), and without warning (dark blue trace), compared to averaged aRT (black trace). All spectral time courses were aligned to the user response onset (thick vertical black line at time 0 s), and the auditory warning for effective- and ineffective-trials were delivered ~5 s before the user response. In the trials following effective auditory warning, the alpha power decreased steadily and reached the averaged aRT in ~7 s. The power spectra remained as low as that of the alert baseline from 7 to 20 s after response onset. In the trials with ineffective auditory warning, the spectral time series fluctuated fierously due to the small number of trials. In the trials without warning, the alpha power fluctuated before response onset and steadily dereased until ~7 s. Thereafter, the alpha power increased again from ~7 to 13 s, suggesting the subjects might be partially arouse by the lane-departure event and their own bebavioral reposense temportally but returned to the fatigue state rapidly thereafter.

## Discussions

Many studies have shown that the brain dynamics correlated with behavioral lapses can be assessed from EEG data. Recent studies have also shown auditory signals can arouse drowsy subjects and affect EEG activities (Lin et al., 2010a, 2013). However, in these studies, the arousing warning was delivered to subjects after they displayed behavioral lapses, which in reality may be too late because the behavioral lapse might have already had catastrophic consequences. Therefore, a system that features real-time lapse detection and delivers warnings to the drowsy subjects is desirable in preventing catastrophic incidents while driving.

The first experiment of this study showed that EEG power changes in either alpha or theta band can be used as an indicator for assessing the subjects' fatigue (cf. Figure 3), and auditory warning temporarily reduces the alpha and theta band power and mitigates the behavioral lapses (cf. Figure 2A). In addition, EEG changes after delivery of auditory warning are a good indicator of the efficacy of arousing warning. More importantly and interestingly, empirical results of the first study showed that arousing auditory signals could always reliably mitigate human behavioral lapses, but these immediate behavioral responses could not guarantee the subjects were fully awake, alert, or attentive, similar to snooze after an alarm is turned off. This finding may open a new research direction of how to accurately confirm a subject's cognitive level for some sustained-attention tasks, such as an aircraft navigator or a long-haul truck driver. In other words, further studies to explore the brain changes in this sleep inertia period may provide valuable insights of brain dynamics during a transitional state of lowered arousal occurring immediately after awakening from sleep. Based on previous studies (Lin et al., 2010a, 2013) and the results of the first experiment, this study further developed a truly OCLDM system to detect/predict cognitive lapse based on the EEG spectra, deliver arousing warning on the occurrence of cognitive lapse, and assess the efficacy of the arousing warning, again, based on the EEG spectra. Most importantly, the EEG spectra changes within ~10 s after delivering arousing warning were closely monitored, such that any false-awake situations could be decreased. This study then documented the design, development, and on-line evaluation of the proposed OCLDM System that featured a lightweight wireless EEG acquisition headgear and a smartphone-based signal-processing platform. Experimental results showed that subjects' EEG power could almost remain at the alert state without bouncing back to the drowsy level (cf. Figure 7). These results suggest that the proposed system could prevent potential behavioral lapses based solely on the EEG signals, and this demonstration could lead to a real-life application of the dry and wireless EEG technology and smartphone-based signal-processing platform. An interesting question is if the neural correlates of fatigue could be generalized across different sustained-attention tasks and different recording conditions. In the past few years, we have conducted several sustained-attention tasks, including auditory target detection tasks (Makeig and Jung, 1995, 1996; Jung et al., 1997), visual compensatory tracking tasks (Huang et al., 2008), and simulated driving tasks (Lin et al., 2005, 2006, 2008b) and found that performance-related EEG dynamics were comparable across tasks (Huang et al., 2007b). Results of these studies also showed the fatigue-related brain dynamics were quite consistent across different recording environments (within a well-controlled EEG laboratory vs. a 6-degree-of-freedom motion platform) and responding methods (using a button press or a steering wheel). Therefore, it is reasonable to believe the methods developed under this study could be translated from laboratory settings to real-world environments.

In sum, this study demonstrated the feasibility of translating a laboratory-based passive BCI system to a neuroergonomic device that is capable of continuously monitoring and mitigating operator neurocognitive fatigue using a pervasive smartphone in real-world environments. The passive BCI technologies might also be applicable to other real-world cognitive-state monitoring, such as attention, distraction, comprehension, confusion, and emotion. We thus believe more real-world passive BCI implementations will emerge in the foreseeable future.

### Conflict of interest statement

The authors declare that the research was conducted in the absence of any commercial or financial relationships that could be construed as a potential conflict of interest.
